# White Matter Network Disruption Is Associated With Melancholic Features in Major Depressive Disorder

**DOI:** 10.3389/fpsyt.2022.816191

**Published:** 2022-04-14

**Authors:** Mengxin He, Yuqi Cheng, Zhaosong Chu, Xin Wang, Jinlei Xu, Yi Lu, Zonglin Shen, Xiufeng Xu

**Affiliations:** ^1^Department of Psychiatry, First Affiliated Hospital of Kunming Medical University, Kunming, China; ^2^Yunnan Clinical Research Center for Mental Disorders, Kunming, China; ^3^Department of Medical Imaging, First Affiliated Hospital of Kunming Medical University, Kunming, China; ^4^Mental Health Institute of Yunnan, First Affiliated Hospital of Kunming Medical University, Kunming, China

**Keywords:** melancholic depression, non-melancholic depression, diffusion tensor imaging, WM network, small-world

## Abstract

**Background:**

The efficacy and prognosis of major depressive disorder (MDD) are limited by its heterogeneity. MDD with melancholic features is an important subtype of MDD. The present study aimed to reveal the white matter (WM) network changes in melancholic depression.

**Materials and Methods:**

Twenty-three first-onset, untreated melancholic MDD, 59 non-melancholic MDD patients and 63 health controls underwent diffusion tensor imaging (DTI) scans. WM network analysis based on graph theory and support vector machine (SVM) were used for image data analysis.

**Results:**

Compared with HC, small-worldness was reduced and abnormal node attributes were in the right orbital inferior frontal gyrus, left orbital superior frontal gyrus, right caudate nucleus, right orbital superior frontal gyrus, right orbital middle frontal gyrus, left rectus gyrus, and left median cingulate and paracingulate gyrus of MDD patients. Compared with non-melancholic MDD, small-worldness was reduced and abnormal node attributes were in right orbital inferior frontal gyrus, left orbital superior frontal gyrus and right caudate nucleus of melancholic MDD. For correlation analysis, the 7th item score of the HRSD-17 (work and interest) was positively associated with increased node betweenness centrality (aBC) values in right orbital inferior frontal gyrus, while negatively associated with the decreased aBC in left orbital superior frontal gyrus. SVM analysis results showed that abnormal aBC in right orbital inferior frontal gyrus and left orbital superior frontal gyrus showed the highest accuracy of 81.0% (69/83), the sensitivity of 66.3%, and specificity of 85.2% for discriminating MDD patients with or without melancholic features.

**Conclusion:**

There is a significant difference in WM network changes between MDD patients with and without melancholic features.

## Introduction

Major depressive disorder (MDD) is a commonly recurring psychiatric disorder with a high disability rate. The clinical features of MDD include persistent depression, decreased vitality, decreased response to external stimuli, and changes in sleeping habits (1). In the DSM, a subtype of MDD is described: pervasive anhedonia (reduction or complete loss of external and inner pleasure experiences), circadian mood fluctuations, guilt, early awakening, psychomotor excitement/ retardation, loss of appetite/weight loss (2). MDD with these highly consistent clinical features is categorized as melancholic MDD (3). Compared with non-melancholic MDD patients, melancholic MDD patients have worse cognitive and social functions (4), higher suicide risk (5), and lower clinical cure rate (4). Melancholic MDD is proposed to be a special subtype of MDD, and this diagnosis may predict treatment efficacy and prognosis (6).

A series of studies have suggested changes in brain function and structure in patients with MDD (7, 8), such as interruption of functional homogeneity and decreased effective connectivity of cortical regions involved in emotion regulation (9). At the same time, many studies have revealed the alterations of brain structure or function in patients with melancholic. For example, its melancholic severity is positively correlated with the orbitofrontal cortex (OFC) activation (10, 11) and negatively correlated with the anterior volume of the caudate nucleus (12). The evaluation of melancholic features may be mainly regulated by OFC (13). Melancholic MDD may have lower mean fractional anisotropy (FA) of OFC (14), and higher FA of the inner capsule of the right forelimb outside the head of the caudate nucleus and inside the lenticular nucleus, than non-melancholic MDD patients (15). However, these studies are only based on the analysis of the function or structure of a single brain area, ignoring the synergy between the various areas of the brain as a network as a whole. The brain network alterations of melancholic MDD are unclear.

Recently, a neuroimaging analysis revealed functional and structural defects in the brains of patients with MDD (16). Research by the Enhancing Neuro Imaging Genetics through Meta-Analysis consortium showed consistent neuroimaging results of MDD brain structure in a multi-site alignment study (17). Functional abnormalities and changes in the cortical structure of non-adjacent brain regions (18), white matter (WM) tract connections between the cortex and subcortical regions between seemingly non-adjacent regions (19), and altered WM tract integrity (disconnection-syndrome) (20) were discovered in patients with MDD. Abnormal transmission of information and nutrients between regions leads to cortical and functional changes in patients with MDD.

Most neuroimaging studies use traditional voxel-based analyses (21), which may not detect subtle and balanced interactions between brain regions or extensive and subtle pathological changes (22). However, the global connection pattern of the brain as well as local connection patterns among brain regions can be evaluated using large-scale network analyses of each brain region (23). This complex brain network analysis (graph theory)—has been widely used in connection group studies of mental disorders (22), such as MDD (24), schizophrenia (25), and bipolar disorder (26), to compare complex brain networks between people with and without these disorders. Small-world network attributes are helpful to explain complex neural connection states between regions (global). The complex brain network of each region (local) can be analyzed through brain networks formed at the connection (edge) between each brain region (node) (27). Compared with voxel-based single brain region analyses, investigating connections between regions may be a more reliable technique for detecting changes in the WM structure in MDD (28), and maybe a helpful method to uncover the pathological mechanism of MDD. In brief, considering the clinical manifestations were different between melancholic depression and non-melancholic depression, the WM structure, which is associated with the pathological mechanism of MDD, may be different between these two subtypes, and associated with different manifestations. We hypothesized that there is a special network model of melancholic depression, the orbitofrontal cortex may play a key role in the network. Thus, we use a complex WM structure brain network based on graph theory to verify this hypothesis. First-episode untreated adult depression patients with and without melancholic characteristics were recruited in our study to avoid the influence of medication.

## Methods

### Participants

Between 2015 and 2017, 82 MDD patients (aged 18–45) were recruited from the outpatient and inpatient departments of the First Affiliated Hospital of Kunming Medical University. Independent diagnoses by at least two professional psychiatrists were conducted according to the structured clinical interviews based on the mood disorders sections of “Structured Clinical Interview for DSM-IV axis I disorders” (SCID-I). Patients were included in this study if they were newly diagnosed with MDD; scored 12 or higher on the Montgomery–Åsberg Depression Rating Scale (MADRS) (29) and 17 or higher on the Hamilton Rating Scale for Depression (HRSD-17) (30); and had no history of taking antipsychotics, undergoing electric shock therapy or psychotherapy, brain injury, or other mental and neurological diseases. Pregnant and left-handed individuals were excluded. Sixty-three healthy controls matched for age, gender, and years of education were also recruited. Healthy patients with a family history of mental illness, any neurological disease, history of mental disorders, drug abuse, or symptoms of mental illness were excluded. It has been approved by the Ethics Committee of Kunming Medical University in Yunnan, China [Ethics Review L No. 50 (2016)]. The study was described in detail to the recruited participants and written informed consent was obtained.

### Subgroups

An M-MDD subgroup was identified based on the DSM description of MDD with melancholic characteristics. Criterion A (at least one item): almost or complete loss of pleasure in all activities or lack of emotional response to pleasant stimuli; criterion B (at least three items): day and night mood changes; extreme guilt; easy to wake up early; psychomotor agitation or retardation; anorexia symptoms or weight loss (2).

The DSM also recommends using the MADRS and HRSD-17 criteria to distinguish between NM- and M-MDD. MADRS criterion A: MADRS 8th item score ≥ 4 (inability to feel), or MADRS 1th or 2th item score ≥ 6 (apparent or reported sadness); concurrent with HRSD-17 criterion B: 1th (depressed mood) or 7th (work and interest) item scores ≥ 3 and at least three of the following: (1) HRSD-17 6th item score ≥ 1 (insomnia-delayed); (2) HRSD-17 8th or 9th item scores ≥ 2 (psychomotor retardation or agitation); (3) HRSD-17 12th or 16th item scores ≥ 2 (anxiety—somatic or loss of weight); (4) HRSD-17 2th item score ≥ 2 (feelings of guilt). Because neither the MADRS nor the HRSD-17 assesses mood changes within 1 day, we were unable to assess diurnal mood variation (31–33).

### Image Acquisition

Magnetic resonance imaging (MRI) was performed using an Achieva 3.0 Tesla MRI system with 16 channels (Philips, Eindhoven, The Netherlands). Diffusion tensor imaging (DTI) was conducted using a single-echo planar imaging sequence in 50 axial planes. DTI scans consist of 32 independent directions, a diffusion weighting factor with non-collinear diffusion sensitization gradient (*b* = 1,000 s/mm^2^), and a reference image without diffusion weighting (b0 image). DTI data were captured using an axial section parallel to the front and rear axis. The imaging parameters were set as follows: TR = 6,800 ms (shortest), TE = 80 ms (shortest), slice thickness = 3 mm (no slice gap), FOV = 230 × 230 mm, matrix size = 116 × 112, voxel size = 2 × 2 × 3 mm, flip angle = 90°, scan time = 8 min 29 s.

### Data Preprocessing

Data preprocessing was performed in MATLAB 2016b using the integrated data processing software PANDA. The preprocessing was performed using the following steps. (1) Correction of head movement and eddy current distortion: registration of the diffusion-weighted image to the b0 image (34). (2) Calculation of the FA to reduce the influence of motion artifacts. (3) Whole-brain fiber bundle imaging: a continuous tracking algorithm for fiber distribution (starting from the deep WM area; voxels with a turning angle >45°; stop tracking at FA <0.15) (35). (4) Matching of participants with WM fiber tract imaging using an automatic anatomical marker segmentation scheme (AAL90) to construct a WM network (36). (5) Assuming that each brain area is regarded as a node, the number of fibers (FN) multiplied by the average FA between the corresponding cortical areas is regarded as the edge weight (w_ij_): w_ij_ = FA_ij_ × FN_ij_ (37, 38). A weighted WM network (90 × 90) was constructed for each participant.

### Network Analysis

Using the GRETNA package (http://www.nitrc.org/projects/gretna/) to perform small-world network operations, healthy human WM networks were found to exhibit the attributes of small-world networks, which are between random networks and regular networks and enable more efficient local specialization and optimally balanced global integration (39). To better understand the characteristics of the small-world network, we analyzed the global attributes (small-worldness) and local attributes (node attributes) (22, 26). Small-worldness includes the normalized clustering coefficient (γ), normalized characteristic path length (λ), characteristic path length, clustering coefficient, global efficiency, and local efficiency (40). Small-worldness (σ) is γ > 1 and λ ≈ 1, or $\sigma =\frac{\gamma }{\lambda }=\frac{\frac{C_{p}^{real}}{C_{p}^{rand}}}{\frac{L_{p}^{real}}{L_{p}^{rand}}}$, where $C_{p}^{rand}$ and $L_{p}^{rand}$ are the averaged values of cluster coefficients and shortest path length of 100 random networks with the same N, V, and degree distribution as the real network (39). We assessed the following six node parameter attributes: node betweenness centrality (aBC), node degree centrality (aDC), node clustering coefficient (aCP), node efficiency (aEfficiency), node local efficiency (aEloc), and node shortest path length (aLP). aBC refers to the number of times a node acts as the shortest bridge between the other two nodes ($B_{nod}\left(i\right)\frac{1}{\left(N-1\right)\left(N-2\right)}\overset{N}{\underset{h=1}{\sum }}\overset{N}{\underset{j=1,h\neq j\neq i}{\sum }}\frac{\rho_{hj\left(i\right)}}{\rho_{hj}}$, where ρ_*hj*(*i*)_ is the total number of the shortest path lengths between nodes *h* and *j*, which pass through *h* for a specific node *i*) (40). Additional details are provided as Supplementary Materials.

### Statistical Analyses

Analysis of variance was performed to analyze group differences in age and years of education using SPSS18.0, and a two-sample *t*-test was used to analyze group differences in MDD and MADRS scores. A chi-square test was performed to describe the gender distribution. The significance level for all tests was *p* < 0.05.

Analysis of covariance (ANCOVA) was performed to assess the small-world network differences among the three groups with gender, age, and years of education as covariates, then *post-hoc* analysis was used to find out the alterations between each group with gender, age, years of education, and MADRS total score as covariates. We tested the topological small-world network attributes using a sparsity threshold of 5% < sparse <50% to reduce the influence of deviation caused by a single threshold. The measurement network was calculated as the area under the entire curve (sparse threshold range). The result was corrected for multiple comparisons using FDR (false discovery rate) to *p* < 0.05 (41, 42).

To assess the node attributes differences among the three groups with gender, age, and years of education as covariates by ANCOVA analysis. And then use *post-hoc* analysis was used to look for changes between each group with gender, age, years of education, and MADRS total score as covariates. To reduce the error in node parameters analysis, non-parametric tests (10,000 times) are used for correction, and the distribution of identification data confirms the application of non-standard test statistics (43), to correct for multiple comparisons, using FDR correction.

GRETNA (http://www.nitrc.org/projects/gretna/) was used to extract relevant values from brain regions with abnormal node attributes. Pearson's correlation analysis was conducted to assess the relationship between anomalous node attributes and HRSD item scores (FDR correction *p* < 0.05).

### Support Vector Machine Analysis

Using LIBSVM software (https://www.csie.ntu.edu.tw/~cjlin/libsvm/), a support vector machine (SVM) was used to classify healthy people and people with MDD, as well as people with non-melancholic MDD and melancholic MDD, based on the anomalous node attributes of the identified abnormal brain regions.

## Results

### Demographic and Clinical Characteristics

Demographic and clinical characteristic data are presented in Table 1. No significant differences in age, gender, or years of education were detected among the three groups, and no statistical difference in illness duration was detected between the two MDD groups. Significant differences in MRADS scores were observed between MDD groups. The MRADS scores of the M-MDD group were significantly higher than those of the NM-MDD group.

**Table 1:** Demographic and clinical characteristics of all participants.

**Variables (mean ±SD)**	**Control (*n* = 63)**	**NM-MDD (*n* = 59)**	**M-MDD (*n* = 23)**	***F*/*t* or χ^2^**	***P*-value**
Handedness (R/L)	63/0	59/0	23/0	-	-
Age (years)	34.3 ± 10.4	33.7 ± 10.3	32.4 ± 11.2	0.68	0.50^a^
Gender (M/F)	38/25	43/16	16/7	2.26	0.32^c^
Education (y)	13.0 ± 4.2	11.7 ± 4.5	11.1 ± 4.4	2.18	0.12^a^
Duration of illness (mo)	-	12.0 ± 17.9	9.3 ±1 2.9	0.77	0.44^b^
MADRS total score	-	28.1 ± 6.4	37.5 ± 4.9	7.0	<0.001^b^
MADRS Item 1th	-	3.0 ± 1.3	4.1 ± 1.1	3.70	0.001^b^
MADRS Item 8th	-	3.3 ± 1.0	4.2 ± 0.42	5.5	<0.001^b^

*SD, standard deviation; MADRS, Montgomery and Asberg Depression Rating Scale; MADRS Item 1th (0–6 score): Apparent Sadness; MADRS Item 8th (0–6 score): Inability to Feel*.

*R, right; L, left; mo, month; M, male; F, female*.

a *The P-values were obtained by ANOVA*.

b *The P-values were obtained by two-sample t-test*.

c *The P values were obtained by chi-square test*.

### Small-Worldness Differences Between Groups

**Figure 1:**
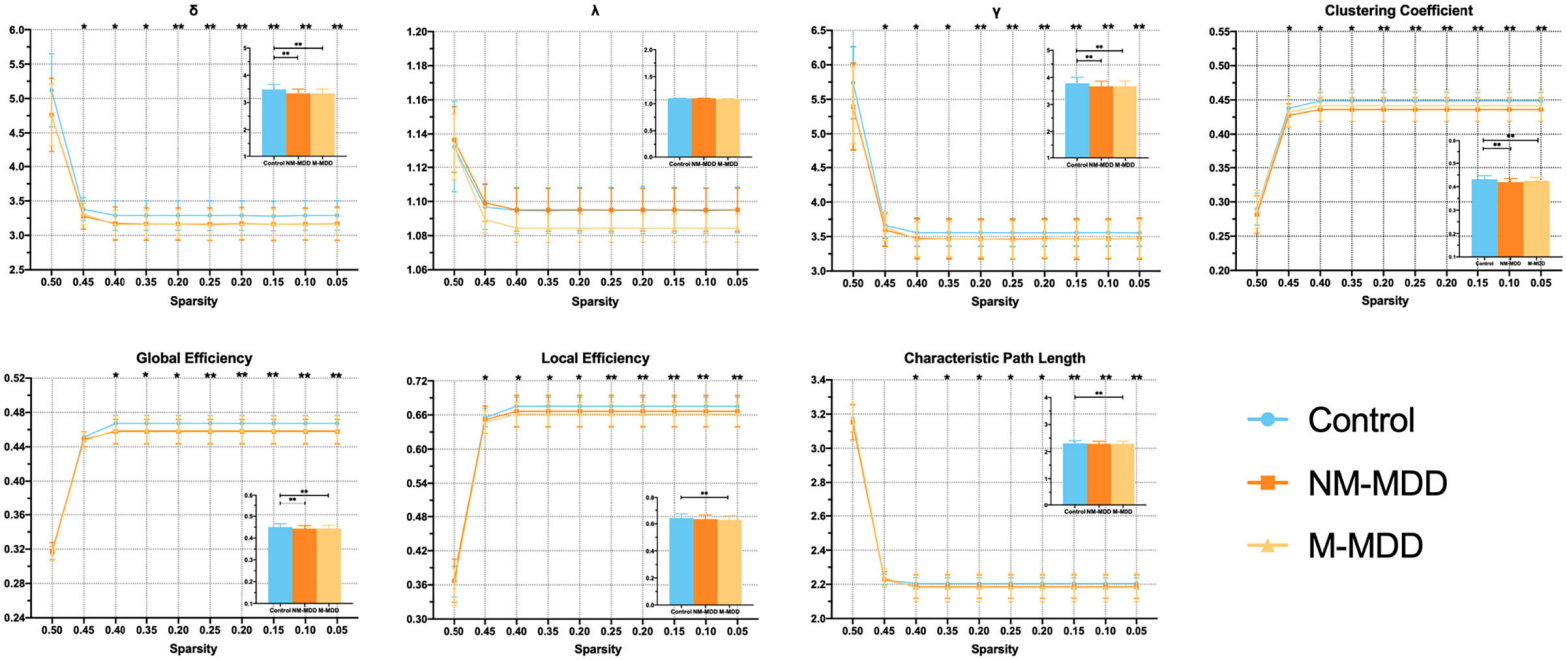
Group comparison of small-world network measures among NM-MDD, M-MDD, and control groups. MDD groups showed significantly decreased σ, γ, clustering coefficient, and global efficiency; M-MDD group showed significantly decreased local efficiency and increased characteristic path length (*p* < 0.05, FDR corrected). σ, small-worldness; λ, normalized characteristic path length; γ, normalized clustering coefficient. ^*^p < 0.05; ^**^p < 0.005.

The ANCOVA analysis with gender, age, and years of education as covariates revealed differences in small-worldness between the three groups. Small-worldness was significantly reduced at the threshold 0.45–0.05 (σ, γ, clustering coefficient, and global efficiency) in the two MDD groups. Compared with the control group, the M-MDD group had significantly reduced local efficiency and significantly increased characteristic path length at the threshold 0.40–0.05 (*p* < 0.05, FDR corrected). No significant difference was found in NM-MDD (Figure 1).

### Node Attributes

#### Node Attribute Differences Between M-MDD, NM-MDD, and Healthy Controls

**Figure 2:**
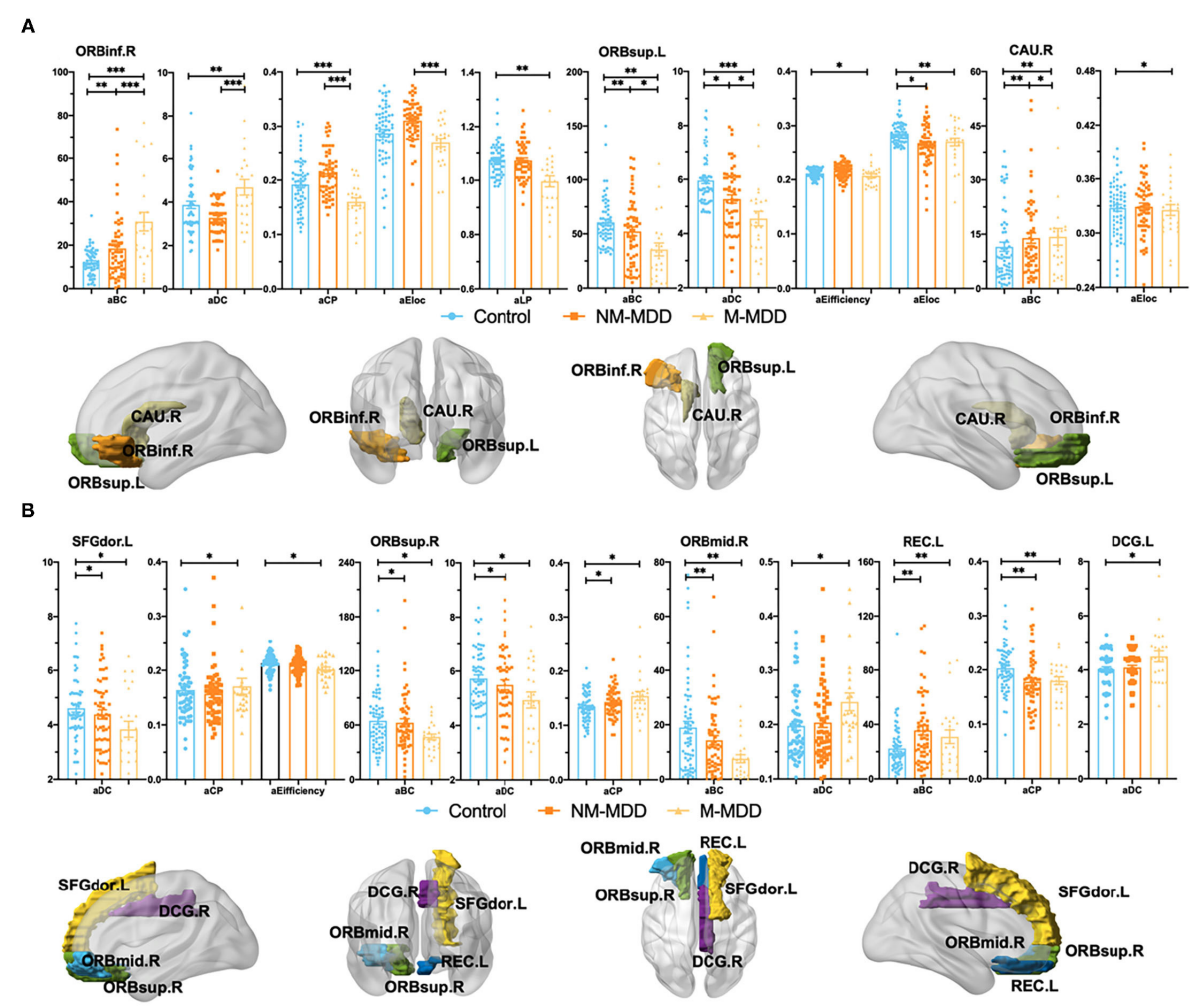
Group comparison of Node attributes among NM-MDD, M-MDD, and control groups. **(A,B)**: MDD groups showed significantly decreased aBC (ORBsup.L, ANG.R, ORBsup.R and ORBmid.R), aDC (ORBsup.L, SFGdor.L and ORBsup.R), aEifficiency (ORBsup.L and SFGdor.L), aCP (ORBinf.R and REC.L), aEloc (ORBsup.L and CAU.R) and increased aBC (ORBinf.R, CAU.R and REC.L), aDC (ORBinf.R, ORBmid.R and DCG.L), and aCP (SFGdor.L and ORBsup.R). **(A)** Group comparison of Node attributes between NM-MDD and M-MDD showed abnormal brain regions in ORBinf.R ORBsup.L and CAU.R. **(B)** Only compared with the control group, MDD groups showed abnormal brain regions SFGdor.L, ORBsup.R,O RBmid.R, REC.L, and DCG.L. FDR corrected, *p < 0.05, **p < 0.005, ***p < 0.0001. ORBinf (Orange, Orbital inferior frontal gyrus) ORBsup (Green, Orbital superior frontal gyrus), CAU (Earthy, Caudate nucleus), SFGdor (Yellow, Dorsolateral superior frontal gyrus), ORBmid (Sky-blue, Orbital middle frontal gyrus) REC (Dark-blue, Rectus gyrus) and DCG (Purple, Median cingulate and paracingulate gyrus); L, Left hemisphere; R, Right hemisphere.

Compared with the control, six abnormal brain regions in NM-MDD group are as follows: right orbital inferior frontal gyrus (ORBinf.R) (increased aBC), left orbital superior frontal gyrus (ORBsup.L) (decreased aBC, aDC, and aEloc), right caudate nucleus (CAU.R) (increased aBC), left dorsolateral superior frontal gyrus (SFGdor.L) (decreased aDC), right orbital superior frontal gyrus (ORBsup.R) (decreased aBC and aDC; increased aCP), right orbital middle frontal gyrus (ORBmid.R) (decreased aBC), and left rectus gyrus (REC.L) (decreased aCP; increased aBC) (Table 2; Figure 2).

**Table 2:** ANCOVA results of nodal parameters differences among patients with healthy groups.

**NM-MDD vs. M-MDD**	**aBC**		**aDC**		**aCP**		**aEiffiency**		**aEloc**		**aLP**	
	***T***	***P***	***T***	***P***	***T***	***P***	***T***	***P***	***T***	***P***	***T***	***P***
ORBinf.R	−5.271	<0.0001^***^	−5.130	<0.0001^***^	5.061	<0.0001^***^	1.869	0.06	−4.653	<0.0001^***^	3.022	0.001^**^
ORBsup.L	2.152	0.019^*^	2.431	0.015^*^	−0.992	0.325	2.370	0.017^*^	0.490	0.621	−0.750	0.457
CAU.R	2.235	0.017^*^	1.452	0.154	0.384	0.712	1.001	0.32	0.503	0.624	−0.883	0.381
**Three groups**	**aBC**		**aDC**		**aCP**		**aEiffiency**		**aEloc**		**aLP**	
	***F***	***P***	***F***	***P***	***F***	***P***	***F***	***P***	***F***	***P***	***F***	***P***
ORBinf.R	13.90	<0.0001^***^	11.74	<0.0001^***^	11.320	<0.0001^***^	2.494	0.086	8.206	0.0004^**^	7.018	0.001^**^
ORBsup.L	7.292	0.0009^**^	15.35	<0.0001^***^	0.587	0.557	4.385	0.014^*^	7.019	0.001^**^	1.536	0.219
CAU.R	5.512	0.004^**^	2.15	0.12	0.744	0.477	0.511	0.601	3.495	0.033^*^	0.491	0.613
SFGdor.L	1.003	0.374	3.27	0.04^*^	5.412	0.005^*^	3.503	0.032^*^	2.248	0.109	2.780	0.065
ORBsup.R	4.521	0.013^*^	3.13	0.04^*^	4.387	0.014^*^	2.764	0.066	2.780	0.065	0.414	0.661
ORBmid.R	5.591	0.004^**^	2.10	0.13	3.653	0.028^*^	2.108	0.125	2.299	0.104	1.561	0.213
REC.L	5.554	0.004^**^	2.48	0.09	5.369	0.006^**^	1.090	0.339	2.881	0.059	0.653	0.522
DCG.L	1.195	0.313	4.02	0.02^*^	0.130	0.878	2.334	0.101	0.622	0.538	2.366	0.097

*ORBinf, Orbital inferior frontal gyrus; ORBsup, Orbital superior frontal gyrus; CAU, Caudate nucleus; SFGdor, Dorsolateral superior frontal gyrus; ORBmid, Orbital middle frontal gyrus; REC, Gyrus rectus; and DCG, Median cingulate and paracingulate gyrus; L, Left hemisphere; R, Right hemisphere*.

*FDR corrected*,

* *p < 0.05*,

** *p < 0.005*,

*** *p < 0.0001. Bold indicates that FDR correction is statistically significant*.

Compared with the control, eight abnormal brain regions in M-MDD group are as follows: ORBinf.R (decreased aCP; increased aBC aDC and aLP), ORBsup.L (decreased aBC, aDC, aEfficiency and aEloc), CAU.R (decreased aEloc; increased aBC), SFGdor.L (decreased aDC and aEfficiency; increased aCP), ORBsup.R (decreased aBC and aDC; increased aCP), ORBmid.R (decreased aBC; increased aDC), REC.L (decreased aCP; increased aBC), and left median cingulate and paracingulate gyrus (DCG.L) (increased aDC) (Table 2; Figure 2).

#### Node Attribute Differences Between NM-MDD and M-MDD

Compared with NM-MDD, in the M-MDD group right orbital inferior frontal gyrus had significantly increased node attributes (aBC and aDC) and decreased attributes (aCP and aEloc). The left orbital superior frontal gyrus had significantly decreased attributes (aBC, aDC, aEfficiency, and aEloc). The right caudate nucleus had significantly decreased node attributes (aBC and aEloc) (Table 2; Figure 2A).

### Correlations Between aBC and Clinical Characteristics

**Figure 3:**
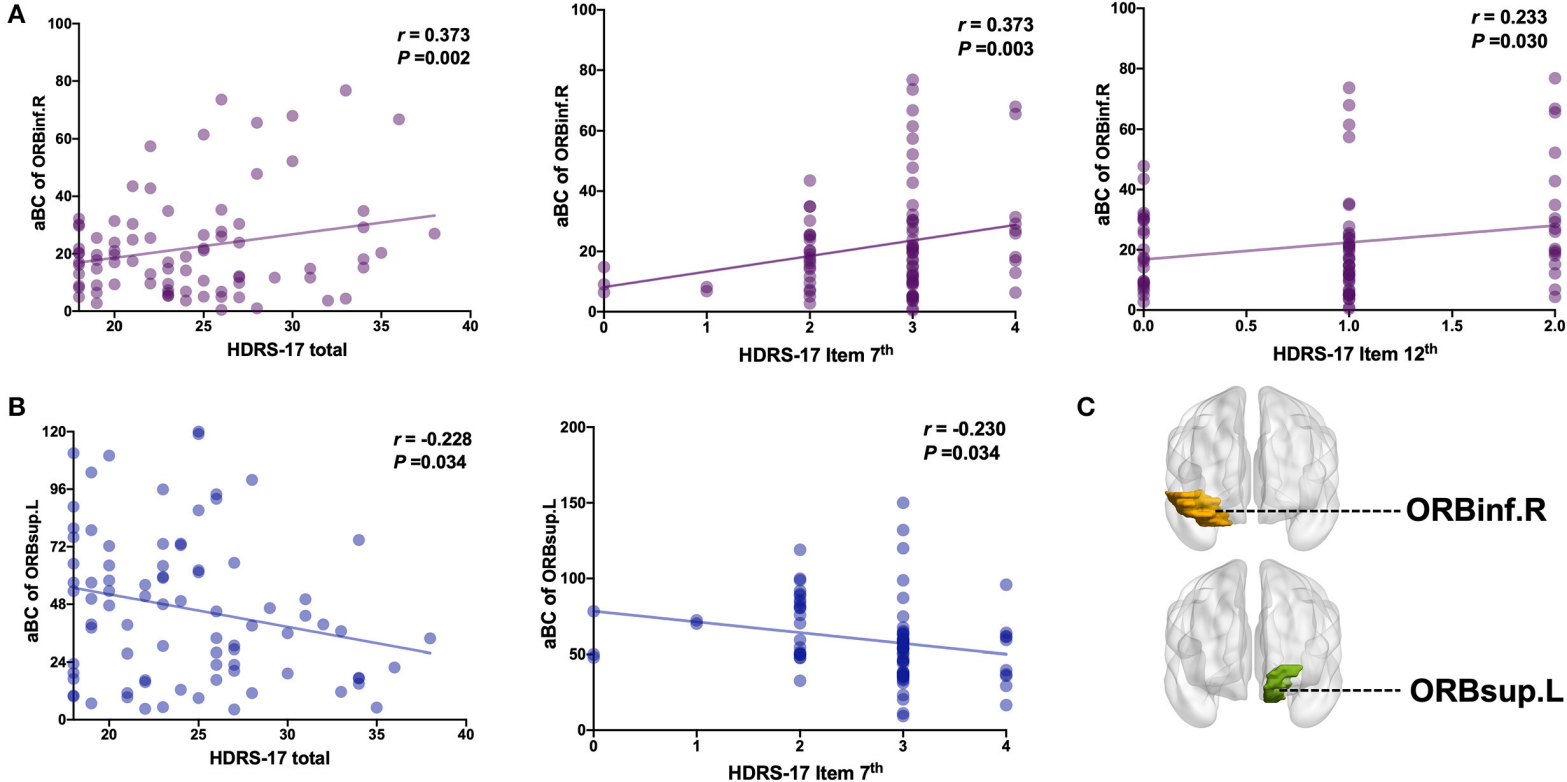
Correlation analysis of HRSD-17 factor scores on aBC of ORBinf.R and ORBsup.L. **(A)** aBC of ORBinf.R is positively correlated with HRSD-17 total score or factor score. **(B)** aBC of ORBsup.L is negatively correlated with HRSD-17 total score or factor score. **(C)** Brain regions visualization. L, Left hemisphere; R, Right hemisphere; ORBinf (Purple, Orbital inferior frontal gyrus); ORBsup (Blue, Orbital superior frontal gyrus). HRSD Item 7th (0–4 score): Reduced Work and Activities; HRSD Item 12th (0–2 score): Gastrointestinal Somatic Symptoms.

Increased aBC in right orbital inferior frontal gyrus was positively correlated with the HRSD-17 total score (*r* = 0.373, *p* = 0.002), 7th item score (work and interest; *r* = 0.373, *p* = 0.003) and 12th item score (gastrointestinal somatic symptoms *r* = 0.233, *p* = 0.030) (Figures 3A,C). Decreased aBC in left orbital superior frontal gyrus was negatively correlated with the HRSD-17 total score (*r* = −0.228, *p* = 0.0034) and 7th item score (*r* = −0.230, *p* = 0.034) (Figures 3B,C). There was no significant difference in other items in HRSD-17 (see the Supplementary Materials for the correlation to the MADRS items).

### SVM Classification Analysis

**Figure 4:**
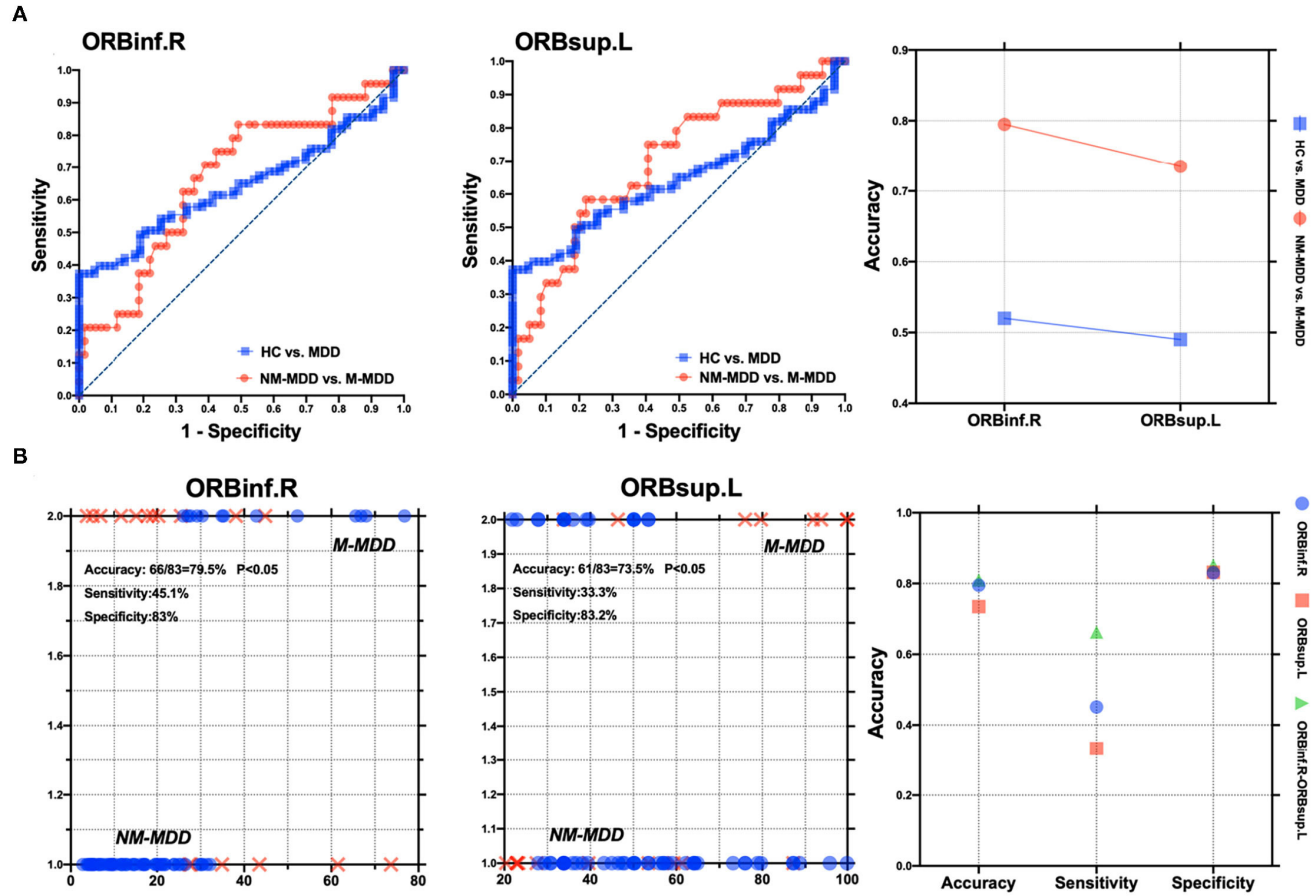
Visualization of classification by using support vector machine (SVM) using the aBC values of ORBinf.R and ORBsup.L. **(A)** Visualization of parameters from healthy controls vs. MDD patients and NM-MDD vs. M-MDD. **(B)** Classification map of aBC values in ORBinf.R and ORBsup.L and SVM parameters as two features. L, Left hemisphere; R, Right hemisphere; ORBinf, Orbital inferior frontal gyrus; ORBsup, Orbital superior frontal gyrus.

The SVM results showed that abnormal right orbital inferior frontal gyrus and left orbital superior frontal gyrus aBC values could distinguish between M-MDD patients and NM-MDD patients with accuracies of 79.5% (66/83) and 73.5% (61/83), sensitivities of 45.1 and 33.3%, and specificities of 83.0 and 83.2%, respectively. The combination of abnormal aBC in right orbital inferior frontal gyrus and left orbital superior frontal gyrus could better distinguish between MDD groups, showing the highest accuracy [81.0% (69/83)], sensitivity (66.3%), and specificity (85.2%). The accuracy, sensitivity, and specificity for distinguishing between healthy and MDD populations were not high (Figure 4).

## Discussion

This study explored the characteristic model of the orbitofrontal lobe WM structure brain network in MDD patients with and without melancholic features. The WM network of MDD patients conforms to the attribute of small-world network, but compared with the healthy control, the global information efficiency of MDD patients is weakened, which refers to the overall efficiency of parallel information transmission in the network (σ, γ, clustering coefficient, and global efficiency) (22) and the node attributes of some brain regions, such as the prefrontal lobe and cingulate gyrus, are ill-conditioned (26). We observed microscopic structural differences in the WM structure network between MDD types. Compared with non-melancholic MDD, the global information efficiency was weaker, and the WM structure network was more random and inefficient in melancholic MDD. Although anomalies of multiple node attributes are observed in the WM network, node betweenness centrality (aBC) is a metric that reflects the importance of a single node, which can better measure the influence of brain regions on information transmission in the network (25). Abnormal aBC was found in almost all abnormal brain regions. In the MDD patients with melancholic features, increased aBC in the right orbital inferior frontal gyrus was positively correlated with the 7th item score of the HRSD-17 (work and interest). It is a measure of the severity of anhedonia, the main characteristic symptom in MDD patients with melancholic features (2). Additionally, decreased aBC in the left orbital superior frontal gyrus was negatively correlated with the 7th item score of the HRSD-17. These results suggest that abnormal aBC in the right orbital inferior and left orbital superior frontal gyri may be the neurobiological features associated with melancholic features.

Compared with healthy controls, patients with MDD have weaker small-world attributes, and the network information transmission order is interrupted, and the efficient information network is transformed into a random network which weakens of information transmission efficiency (26). Additionally, the melancholic MDD group had a lower local efficiency (aEloc) and longer node shortest path length (aLP) than HC. Although the difference between the two MDD subgroups is not obvious, this trend shows that the small-world network attribute of the melancholic subtype is developing more disorderly (24).

The orbitofrontal cortex believed that it is mostly related to the problems of cognition (44) and pleasure experience (information processing ability and response to external stimuli) (45). The caudate nucleus associated with rewarding process and pleasure experience (12). Our study found that compared with non-melancholic MDD and healthy controls, the increased aBC were found in right inferior orbital frontal gyrus and right caudate nucleus while the decreased aBC was found in left superior orbital superior frontal gyrus in melancholic MDD patients group. This can explain the more serious cognitive and pleasure experience problems experienced in melancholic MDD (low information processing ability and weaker response to external stimuli) (4). Therefore, we can speculate that the increase of aBC in the right inferior orbital frontal gyrus and the right caudate nucleus, and the decrease of aBC in the left superior orbital frontal gyrus may be the stable and unique neurobiological characteristics of melancholic MDD.

Compared with non-melancholic MDD patients, the abnormal network betweenness centrality in right inferior orbital frontal gyrus, the left orbital superior frontal gyrus and the right caudate nucleus was found in MDD patients with melancholic featured. Node betweenness centrality (aBC) refers to the number of times a node acts as the shortest bridge between the other two nodes. The abnormal aBC of two regions indicates that the information transmission balance is disturbed (46). The right inferior orbital frontal gyrus and the left superior orbital frontal gyrus belong to the orbitofrontal cortex (47). The caudate nucleus is part of the striatum (46). Previous study has shown the dysfunction and structure deficits of orbital frontal lobe were associated with the decision/reward deficits, lack of emotion/behavior control, and negative cognition in MDD patients (41), as well as the reward neural circuits are affected by the prefrontal–striatal pathway (48). The caudate nucleus (49), and orbital frontal lobe (48) are involved in the process of emotional and cognitive regulation. When MDD patients process positive emotional task information, the severity of anhedonia is related to the activation of the orbital frontal lobe and is negatively related to the volume of the anterior caudate nucleus (31). A study of brain function in depression with loss of appetite reported enhanced activation of the right orbital frontal lobe in the reward loop (50). Therefore, the interruption of these brain regions transmitting information may lead to severe anhedonia, negative cognition, and loss of appetite (weight loss) in melancholic depression (49). This may be the neuropathological basis for the obvious and poorer cognitive performance of melancholic MDD compared with HC or non-melancholic depression (51).

The right orbit inferior frontal gyrus aBC was positively correlated with the HRSD-17 total score, 7th item (work and interest), and 12th item score (gastrointestinal somatic symptoms). The left orbit superior frontal gyrus aBC was negatively correlated with the HRSD-17 total score and 7th item score. non-melancholic and melancholic MDD subtypes were grouped according to strict criteria (A and B) while meeting the requirements of two groups (32). The significant difference in the orbitofrontal gyrus between the two MDD groups may explain the more severe anhedonia experienced and loss of appetite in non-melancholic MDD. The main symptoms of melancholic MDD are anhedonia and reduced or absent internal and external reaction emotions (3), as well as more severe cognitive deficits than non-melancholic MDD (4), it also showed a significant loss of appetite (3), which may be due to a weakened response to external/internal stimuli. This may be the main reason for the obvious difference between the two MDD subtypes (6). Therefore, we speculate that abnormal aBC in the right orbital inferior frontal gyrus and left orbital superior frontal gyrus may be potential imaging markers for distinguishing melancholic MDD from non-melancholic MDD.

SVMs are widely used in biomedical research to diagnose severe mental illnesses (52), such as major depression, schizophrenia, and bipolar disorder (53–55). Generally, the accuracy, sensitivity, and specificity are not below 65%, indicating fair reliability. When these parameters are >70%, the SVM can be used as a reliable diagnostic index (56, 57). In this study, the SVM analysis showed that the melancholic and non-melancholic MDD groups could be distinguished based on aBC abnormalities of either the right orbital inferior frontal gyrus or the left orbital inferior frontal gyrus with accuracies >70% but low sensitivities. However, by combining the right orbital inferior frontal gyrus and left orbital inferior frontal gyrus aBC values, the SVM could distinguish the MDD subtypes with high accuracy (81.0%), sensitivity (66.3%), and specificity (85.2%). Therefore, the combination of the right orbital inferior frontal gyrus and left orbital inferior frontal gyrus aBC values can be used as a reliable biomedical indicator to distinguish MDD patients with and without melancholic features.

Compared with non-melancholic MDD, melancholic MDD is characterized by worsened clinical symptoms, including universal anhedonia, mood, cognition, and loss of appetite (3). There are other subtypes of non-melancholic MDD patients, with other characteristics (such as anxiety, atypical sleep disorders, physical symptoms, etc.) rather than melancholic MDD patients (6). This may be the right inferior orbital frontal gyrus and the left superior orbital frontal gyrus can only be used as one of the reasons to distinguish between non-melancholic and melancholic depression. Therefore, there can be a misfit when distinguishing between health and depression. To improve diagnosis accuracy, future applications should focus not only on single voxel-based brain imaging technology (7, 8) but also on the relationships between brain regions and multi-modal brain network technology (58), combining functional and structural brain network data (40).

As shown in our series of studies, melancholic depression can be classified as a special subtype of depression (6). Future research could further explore the different subtypes of depression. Imaging analysis based on the WM structure of the brain network provides an objective basis for distinguishing between melancholic and non-melancholic depression and provides evidence that different subtypes of depression may have different neuropathological mechanisms.

## Conclusion

In the WM structure network of MDD patients, the information transmission efficiency is lower and more disordered. The differences in WM structure in the MDD patient group were mainly in the orbitofrontal gyrus and cingulate gyrus regions. Our results demonstrate that MDD patients with melancholic depression have a unique WM structure network pattern that differentiates them from patients with non-melancholic features. The WM structure network in melancholic depression is more biased toward random networks and less efficient. aBC defects in the right orbital inferior and left orbital superior frontal gyri may be stable and unique neurobiological features of melancholic depression, representing potential imaging markers for distinguishing melancholic and non-melancholic depression subtypes.

## Limitations and Future Directions

An important limitation of this study is its cross-sectional design; longitudinal research is required to confirm our findings. Changes in the WM network structure are not as sensitive as changes in a single structure and function, and further large-scale research and evidence are needed. Although the number of patients with melancholic depression is small, our results also provide a certain reference value for distinguishing melancholic depression from non-melancholic depression. In the future, we can expand the sample size to verify the results of this study. Melancholic and non-melancholic dependence were distinguished using an objective scale evaluation. The questionnaire of the supervisor is not used for the distinction. In the future, it can be verified in the sample using questionnaires to verify the reliability of the results of this study. Additionally, MDD subtypes may be related to the prognosis; therefore, long-term follow-up studies are needed to evaluate differences in treatment outcomes between patients with different subtypes. Future research should explore and verify the multi-modal brain network using larger cohorts and datasets.

## Data Availability Statement

The original contributions presented in the study are included in the article/Supplementary Materials, further inquiries can be directed to the corresponding authors.

## Ethics Statement

The studies involving human participants were reviewed and approved by First Affiliated Hospital of Kunming Medical University [Ethics Review L No. 50 (2016)]. The patients/participants provided their written informed consent to participate in this study.

## Author Contributions

All authors provided have made strong, direct, effective contributions to this research, and agree to publish it.

## Funding

This study was funded by Yunnan Basic Research Projects-Union Foundation [2019FE001(-144)], Yunnan Clinical Research Center for Mental Disorders (202102AA100058), National Natural Science Foundation of China (81660237), Yunnan Health Training Project of High Level Talents (H-2018090), and Yunnan Provincial Health Institute Research Project (2018NS0109).

## Conflict of Interest

The authors declare that the research was conducted in the absence of any commercial or financial relationships that could be construed as a potential conflict of interest.

## Publisher's Note

All claims expressed in this article are solely those of the authors and do not necessarily represent those of their affiliated organizations, or those of the publisher, the editors and the reviewers. Any product that may be evaluated in this article, or claim that may be made by its manufacturer, is not guaranteed or endorsed by the publisher.
