# Supplementary material for: White Matter Network Disruption Is Associated With Melancholic Features in Major Depressive Disorder
Source: Front Psychiatry. 2022 Apr 14;13:816191. doi: 10.3389/fpsyt.2022.816191 (PMC9046786; doi:10.3389/fpsyt.2022.816191)
Supplement: Supplementary file 1 [file Data_Sheet_1.docx]

**supplementary material**

** Supplementary Table1. Procedure of the study**

**Supplementary Table2. Introduction of topological measurements (including five global network metrics and six nodal metric) and their meaning in structural brain networks.**

|  | **Network properties** | **Definitions** |
| --- | --- | --- |
| Global characteristics | characteristic path length (Lp) | the average minimal travel distance  between nodes in the network. It represents the global  integration of the network |
|  | clustering coefficient (Cp) | the extent of a local density or cliquishness of the network. |
|  | global efficiency (E_glob_) | Eglobal is a measure of the global efficiency of parallel information transfer in the network. It is inversely related to Lp. |
|  | local efficiency (E_loc_) | Elocal is a measure of the information exchange at the clustering level. |
|  | Small-worldness (σ) | 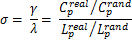is a scalar measurement of the small-world w rand rand property of a network, where γ and λ denote the average γ and λ of an ensemble of 100 surrogate random networks. A small-world network has high local clustering and short paths between brain regions. |
| Nodal characteristics | nodal Degree Centrality (aDC) | The nodal degree for a given node reflects its information communication ability in the functional network. |
|  | nodal efficiency (aEfficiency) | Nodal efficiency is the inverse of the harmonic mean of the shortest path length between node i and all other nodes. A region with high nodal efficiency indicates great interconnectivity with other regions in the network. |
|  | Nodal local efficiency (aEloc) | The local efficiency for a given node measures how efficient the communication is among the first neighbors of this node when it is removed. |
|  | nodal shortest path length(aLP) | The shortest path length of a given node quantifies the mean distance or routing efficiency between this node and all the other nodes in the network. |
|  | nodal clustering coefficient(aCP) | The clustering coefficient of a given node measures the likelihood its neighborhoods are connected to each other. |
|  | Nodal betweenness centrality (aBC) | Nodal betweenness centrality refers to the number of times a node acts as the shortest bridge between the other two nodes. |

| **Supplementary Table 3 Brain regions included in the revised Automated Anatomical Labeling (AAL90) atlas.** | | | | | | |
| --- | --- | --- | --- | --- | --- | --- |
| Index | Label | abbreviation | | Index | Label | abbreviation |
| 1 | Precentral | PreCG | | 24 | Lingual | LING |
| 2 | Frontal_Sup | SFGdor | | 25 | Occipital_Sup | SOG |
| 3 | Frontal_Sup_Orb | ORBsup | 26 | | Occipital_Mid | MOG |
| 4 | Frontal_Mid | MFG | | 27 | Occipital_Inf | IOG |
| 5 | Frontal_Mid_Orb | ORBmid | | 28 | Fusiform | FFG |
| 6 | Frontal_Inf_Oper | IFGoperc | | 29 | Postcentral | PoCG |
| 7 | Frontal_Inf_Tri | IFGtriang | | 30 | Parietal_Sup | SPG |
| 8 | Frontal_Inf_Orb | ORBinf | | 31 | Parietal_Inf | IPL |
| 9 | Rolandic_Oper | ROL | | 32 | SupraMarginal | SMG |
| 10 | Supp_Motor_Area | SMA | | 33 | Angular | ANG |
| 11 | Olfactory | OLF | | 34 | Precuneus | PCUN |
| 12 | Frontal_Sup_Medial | SFGmed | | 35 | Paracentral obule | PCL |
| 13 | Frontal_Mid_Orb | ORBsupmed | | 36 | Caudate | CAU |
| 14 | Rectus | REC | | 37 | Putamen | PUT |
| 15 | Insula | INS | | 38 | Pallidum | PAL |
| 16 | Cingulum_Ant | ACG | | 39 | Thalamus | THA |
| 17 | Cingulum_Mid | DCG | | 40 | Heschl | HES |
| 18 | Cingulum_Post | PCG | | 41 | Temporal_Sup | STG |
| 19 | Hippocampus | HIP | | 42 | Temporal_Pole_Sup | TPOsup |
| 20 | ParaHippocampal | PHG | | 43 | Temporal_Mid | MTG |
| 21 | Amygdala | AMYG | | 44 | Temporal_Pole_Mid | TPOmid |
| 22 | Calcarine | CAL | | 45 | Temporal_Inf | ITG |
| 23 | Cuneus | CUN | |  |  |  |

Note:45 brain regions were extracted from the right and left hemispheres, separately, and eight vermis to provide 90 regions in total for each subject.

**Supplementary Table 4 Scale items used to assess melancholic features**

| **DSM melancholic features in MDD** | **Scale items** |
| --- | --- |
| **Criterion A: Montgomery–Åsberg Depression Rating Scale** |  |
| - Lack of reactivity to normally pleasurable stimuli (one of score ≥ 6): | 1. Observed despondency/despair in facial expressions,speech, posture; rated by depth and inability to brighten |
|  | 2. Subjectively reported depressed mood; rated by intensity, duration, and lack of reaction to external events |
| - Loss of pleasure in all or most activities( item score ≥ 4) | 8. Reduced interest in surroundings or normally pleasurable  activities; lack of emotional reaction to circumstances |
| **Criterion B: Hamilton Rating Scale for Depression-17** |  |
| - **main symptoms (one of score ≥ 3):** | 1. depressed mood |
|  | 7. work and interest |
| - **Other symptoms(at least three of):** |  |
| - Excessive guilt | 2. Feelings of guilt |
| - Early-morning awakening | 6. Late insomnia |
| - Psychomotor agitation or retardation | 8. Slowness of thinking, speech, and activity |
|  | 9. Agitation and restlessness |
| - Anorexia or weight loss | 12. Loss of appetite |
|  | 16. Weight loss |

Note. The DSM also recommends using the MADRS and HRSD-17 criteria to distinguish between NM- and M-MDD. MADRS criterion A: MADRS 8th item score ≥ 4 (inability to feel), or MADRS 1 ^th^ or 2 ^th^ item score ≥ 6 (apparent or reported sadness); concurrent with HRSD-17criterion B: 1^th^ (depressed mood) or 7 ^th^ (work and interest) item scores ≥ 3 and at least three of the following: (1) HRSD-17 6 ^th^ item score ≥ 1 (insomnia-delayed); (2) HRSD-17 8 ^th^ or 9 ^th^ item scores ≥ 2 (psychomotor retardation or agitation); (3) HRSD-17 12^th^ or 16 ^th^ item scores ≥ 2 (anxiety – somatic or loss of weight); (4) HRSD-17 2 ^th^ item score ≥ 2 (feelings of guilt). Because neither the MADRS nor the HAMD-17 assesses mood changes within one day, we were unable to assess diurnal mood variation.

**Supplementary Figure**
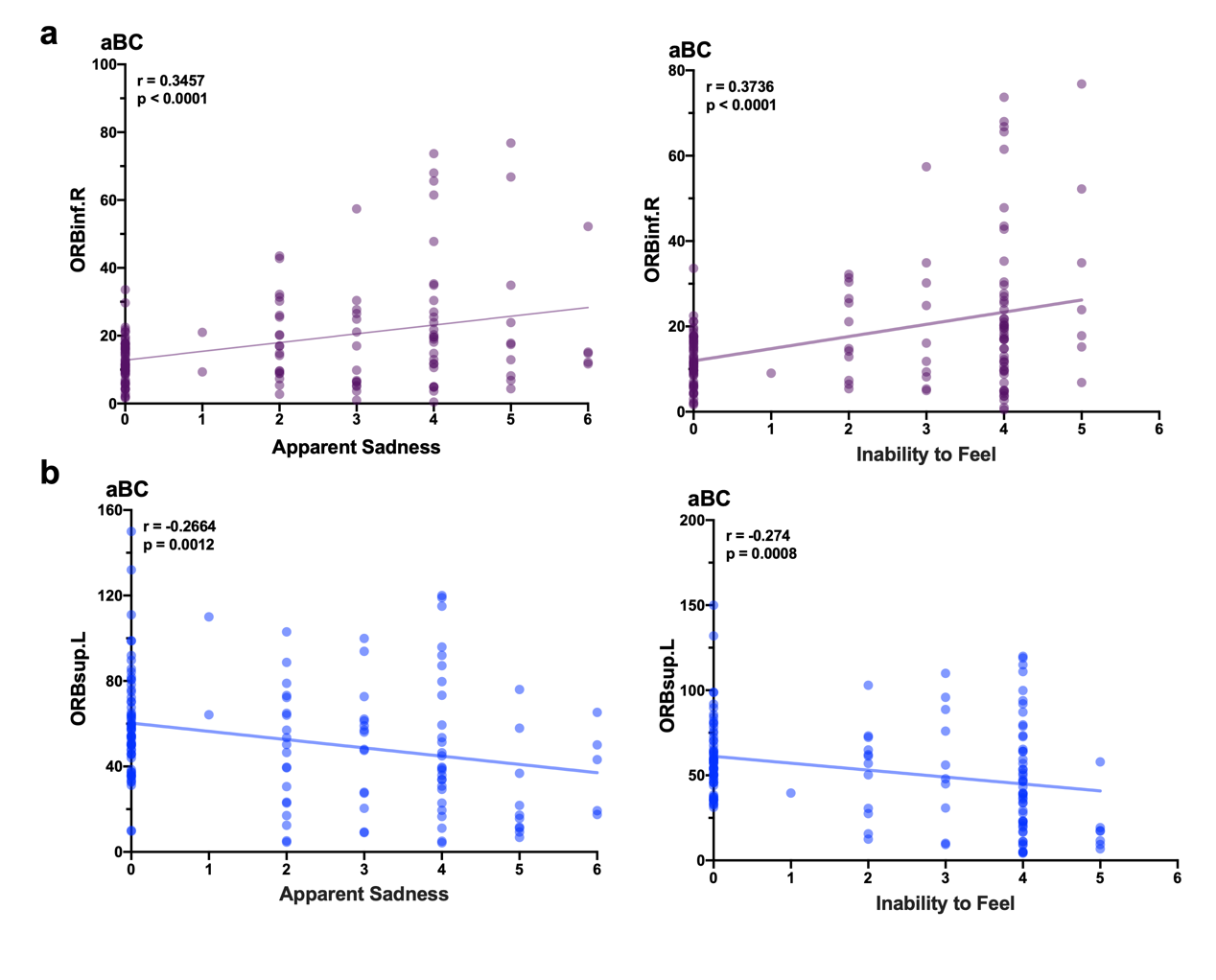
**1 Correlation analysis of MADRS scores on aBC of ORBinf.R and ORBsup.L.**

a: aBC of ORBinf.R is positively correlated with Apparent Sadness and Inability to Feel scores.

b: aBC of ORBsup.L is negatively correlated with Apparent Sadness and Inability to Feel scores.

L: Left hemisphere; R: Right hemisphere; ORBinf (Orbital inferior frontal gyrus); ORBsup (Orbital superior frontal gyrus).

Apparent Sadness: **MADRS** Item 1^th^ (0-6 score); Inability to Feel **MADRS** Item 8^th^ (0-6 score).
